# New therapeutic approaches in psychiatry: contribution of neuroscience

**DOI:** 10.3402/snp.v6.31403

**Published:** 2016-03-17

**Authors:** Virginie Moulier

**Affiliations:** Unité de Recherche Clinique, EPS Ville Evrard, Paris, France

Since the second half of the twentieth century, drug therapy has revolutionized the treatment and prognosis of psychiatric patients. However, a large number of patients experience treatment resistance, which underlines the importance of developing new therapeutic approaches. The early twenty-first century has been marked by significant progress in neurosciences, particularly in neuroimaging, which have facilitated highlighting of the brain and/or cognitive dysfunction among psychiatric patients. These advances have led to the development or updating of ‘new therapeutic neuroscience-based approaches in psychiatry’, complementary to pharmacological treatment.

In this *Socioaffective Neuroscience & Psychology* special issue, we focus on several non-invasive approaches: (1) repetitive transcranial magnetic stimulation (rTMS), the basic principle of which is the application of magnetic pulses to a specific area of the brain through an electromagnetic coil; (2) transcranial direct current stimulation (tDCS), involving the application of a weak electrical current over the scalp; and (3) cognitive remediation, psychosocial care involving cognitive functions training. These three techniques seek to influence neural and/or cognitive function in order to improve psychiatric symptoms. However, mechanisms underlying their clinical efficacy remain unclear. Therefore, this special issue aims at developing a clearer understanding of these new tools. To achieve this goal, Moulier et al. (2016) studied the impact of the rTMS protocol – usually used for treating depression – in 20 healthy subjects, in order to investigate its effects on mood and emotion processing. Based on neuroimaging studies, Isaac and Januel (2016) and Baeken, Brunelin, Duprat, and Vanderhasselt (2016) provided a review of the neural correlates of cognitive remediation and tDCS, respectively.

All these articles should help improve upcoming treatment protocols. These therapeutic tools have the advantage of being non-invasive, fairly easy to use, rarely contraindicated, and inducing no (or very few) side effects. These therapeutic strategies could play an exponential role in future clinical psychiatric practices. Nevertheless, quite a few parameters still need to be defined according to the patient's mental disorder and his or her symptomatology. Further research on these new therapeutic neuroscience-based approaches is therefore required in the field of psychiatry.

*Virginie Moulier,PhD* Unité de Recherche Clinique, EPS Ville Evrard Paris, France
